# High Levels of Exosomes Expressing CD63 and Caveolin-1 in Plasma of Melanoma Patients

**DOI:** 10.1371/journal.pone.0005219

**Published:** 2009-04-17

**Authors:** Mariantonia Logozzi, Angelo De Milito, Luana Lugini, Martina Borghi, Luana Calabrò, Massimo Spada, Maurizio Perdicchio, Maria Lucia Marino, Cristina Federici, Elisabetta Iessi, Daria Brambilla, Giulietta Venturi, Francesco Lozupone, Mario Santinami, Veronica Huber, Michele Maio, Licia Rivoltini, Stefano Fais

**Affiliations:** 1 Unit of Antitumor Drugs, Department of Therapeutic Research and Medicines Evaluation, Istituto Superiore di Sanità, Rome, Italy; 2 Unit of Molecular and Cellular Imaging, Istituto Superiore di Sanità, Rome, Italy; 3 Division of Medical Oncology and Immunotherapy, Department of Oncology, University Hospital of Siena, Istituto Toscano Tumori, Siena, Italy; 4 Unit of Experimental Immunotherapy, Department of Cell Biology and Neurosciences, Istituto Superiore di Sanità, Rome, Italy; 5 Unit of Melanoma and Sarcoma, Fondazione IRCCS Istituto Nazionale Tumori, Milan, Italy; 6 Unit of Immunotherapy of Human Tumours, Fondazione IRCCS Istituto Nazionale Tumori, Milan, Italy; 7 Unit of Cancer Bioimmunotherapy, Department of Medical Oncology, Centro di Riferimento Oncologico IRCCS, Aviano, Italy; Karolinska Institutet, Sweden

## Abstract

**Background:**

Metastatic melanoma is an untreatable cancer lacking reliable and non-invasive markers of disease progression. Exosomes are small vesicles secreted by normal as well as tumor cells. Human tumor-derived exosomes are involved in malignant progression and we evaluated the presence of exosomes in plasma of melanoma patients as a potential tool for cancer screening and follow-up.

**Methodology/Principal Findings:**

We designed an in-house sandwich ELISA (Exotest) to capture and quantify exosomes in plasma based on expression of housekeeping proteins (CD63 and Rab-5b) and a tumor-associated marker (caveolin-1). Western blot and flow cytometry analysis of exosomes were used to confirm the Exotest-based findings. The Exotest allowed sensitive detection and quantification of exosomes purified from human tumor cell culture supernatants and plasma from SCID mice engrafted with human melanoma. Plasma levels of exosomes in melanoma-engrafted SCID mice correlated to tumor size. We evaluated the levels of plasma exosomes expressing CD63 and caveolin-1 in melanoma patients (n = 90) and healthy donors (n = 58). Consistently, plasma exosomes expressing CD63 (504±315) or caveolin-1 (619±310) were significantly increased in melanoma patients as compared to healthy donors (223±125 and 228±102, respectively). While the Exotest for CD63+ plasma exosomes had limited sensitivity (43%) the Exotest for detection of caveolin-1+ plasma exosomes showed a higher sensitivity (68%). Moreover, caveolin-1+ plasma exosomes were significantly increased with respect to CD63+ exosomes in the patients group.

**Conclusions/Significance:**

We describe a new non-invasive assay allowing detection and quantification of human exosomes in plasma of melanoma patients. Our results suggest that the Exotest for detection of plasma exosomes carrying tumor-associated antigens may represent a novel tool for clinical management of cancer patients.

## Introduction

Exosomes are small endosome-derived vesicles (50–100 nm in size), actively secreted through an exocytosis pathway normally used for receptor discharge and intercellular cross-talk [1]–[3]. In addition to major histocompatibility complex proteins (MHC I, MHC II) and proteins involved in antigen presentation, exosomes may carry membrane and cytosolic proteins involved in many cellular functions [1], [4]. These structures are secreted under specific physiological conditions from different cell types such as dendritic cells (DC), lymphocytes, mast cells and epithelial cells [5]–[8]. However, release of exosomes from tumor cells is dramatically increased and represents a constitutive process, often associated with immunosuppressive effects [3], [9], [10].

The role of tumor exosomes in cancer progression is recently emerging, although initial data pointing at these organelles as carriers of tumor antigenic material for DC-mediated T cell cross-priming have supported clinical attempts to use tumor exosomes as anti-cancer vaccines [11]. However, growing evidence concerning a vast array of suppressive effects exerted by these microvesicles on different components of the immune system is clearly supporting the involvement of tumor exosomes in disease progression [3], [12]. In particular, we and others have recently shown that exosomes secreted by human tumor cells of various origins are able to induce apoptosis in activated T cells, through the expression of death ligands (e.g. FasL, TRAIL) [9], [10], [13], inhibit NK functions [14], [15] and promote the generation of myeloid-derived suppressor cells from normal monocytes [10]. These data, together with the reproducible evidence that exosomes of likely tumor origin can be abundantly found in plasma and neoplastic effusions of cancer patients [16]–[18] support a role of tumor exosomes in molding host microenvironment to allow tumor growth and progression [19], [20].

However, the study of the in vivo role of tumor exosomes has been so far penalized by the lack of suitable methods to quantify exosomes from human body fluids, particularly from plasma of cancer patients. The aim of our study was thus to provide a method to detect and quantify exosomes from small amount of human plasma, with the final goal of identifying a tool for assessing the role of tumor exosomes as potential tumor marker and prognostic factor. This might be particularly relevant in melanoma patients, in which sensitive and reliable serum markers are unfortunately still limited while serum LDH (lactate dehydrogenase) levels remain the only prognostic serum factor for assessing disease course and prognosis [21], [22]. Here, we describe an in-house ELISA that allows quantification and characterization of exosomes from different samples, including plasma from tumor-bearing animals and melanoma patients, as well as from tumor cell culture supernatants. These findings suggest that the detection of tumor exosomes in plasma of cancer patients may represent a potential biomarker in the clinical monitoring of tumor malignancies, in particular melanoma.

## Results

### Set up of in-house ELISA for exosomes quantification

#### Exosomes detection in vitro

**Figure 1:**
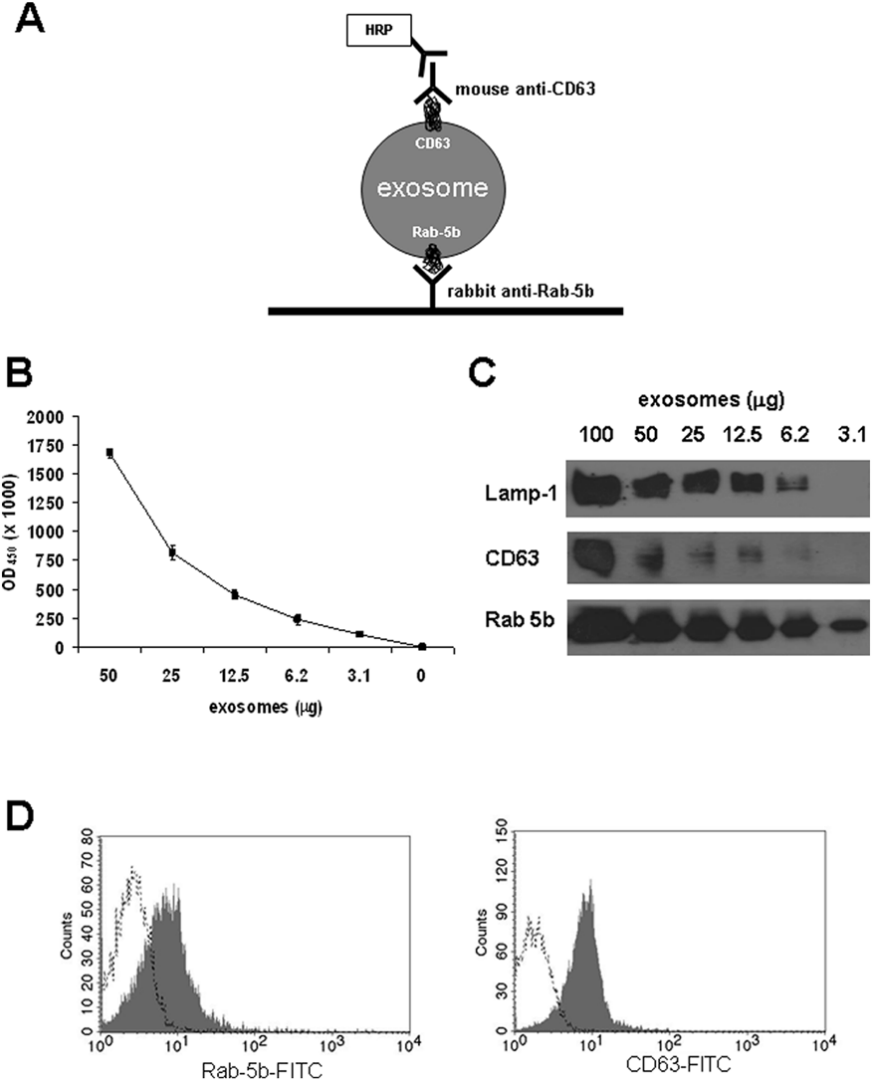
Detection of exosomes purified from cell culture supernatants of human melanoma cells. (A) Schematic representation of the ELISA (Exotest) set up for exosomes detection and quantification. (B) Dose-escalation analysis of purified CD63+ exosomes by Exotest. The initial concentration corresponded to 50 µg of exosomes and exosomes were added in two-fold dilutions. (C) Western blot analysis of CD63, Rab-5b and Lamp-1 expression in different amount of exosomes purified from culture supernatants of human melanoma cells (Me501). (D) FACS analysis of Rab-5b and CD63 expression on melanoma-derived exosomes purified from the supernatant of Me501 cells and coated to latex beads.

The ELISA we developed is based on the presence on exosome of proteins shared with cytoplasmic organelles such as endosomes and lysosomes (Rab-5b and CD63), whose membranes are not shed or recycled as for plasma membrane structures, thus excluding the possible presence of structures deriving from membranes shedding and disruption [23]. Culture supernatants of melanoma cells Me501 and MeBS were processed to obtain purified exosomes and data are shown for Me501 cells. The Exotest (Figure 1A) was able to provide a quantification of the exosomes present in cell culture supernatants, being CD63+ exosomes detectable in a dose-dependent manner (Figure 1B). The negative controls, represented by fractions derived from pellet obtained after the 10000 g centrifugation, exosomes purified from cell culture medium alone and by the only secondary antibody resulted in a barely measurable optical density (OD = 0.07±0.01). Intra and inter-test variability were calculated on six replicates of the same preparation run on three different plates and were 30% and 25%, respectively. A patent application has been recently registered for this in-house ELISA (#US 12/321,412; PCT/EE2009/000001) and the Exotest is currently being standardized to reach variability comparable to commercially available kits (HansaBiomed, O.U., Estonia). Western blot and FACS analysis of the same purified exosome preparations confirmed the data obtained by Exotest. In fact, Rab-5b, Lamp-1 and to a lesser extent CD63 proteins were detectable by WB (Figure 1C) and both Rab-5b and CD63 were also detected by FACS on exosomes bound to latex beads (Figure 1D). However, exosomes detection and quantification by Exotest showed a higher sensitivity for the detection of CD63 protein with respect to WB analysis (Figure 1C). Indeed, while at least 12.5 µg of exosome proteins were needed to properly detect both CD63 and Rab-5b by WB, the Exotest was able to detect exosomes starting from a minimum amount of 3 µg of purified samples.

#### Plasma exosomes in SCID mice engrafted with human melanoma

**Figure 2:**
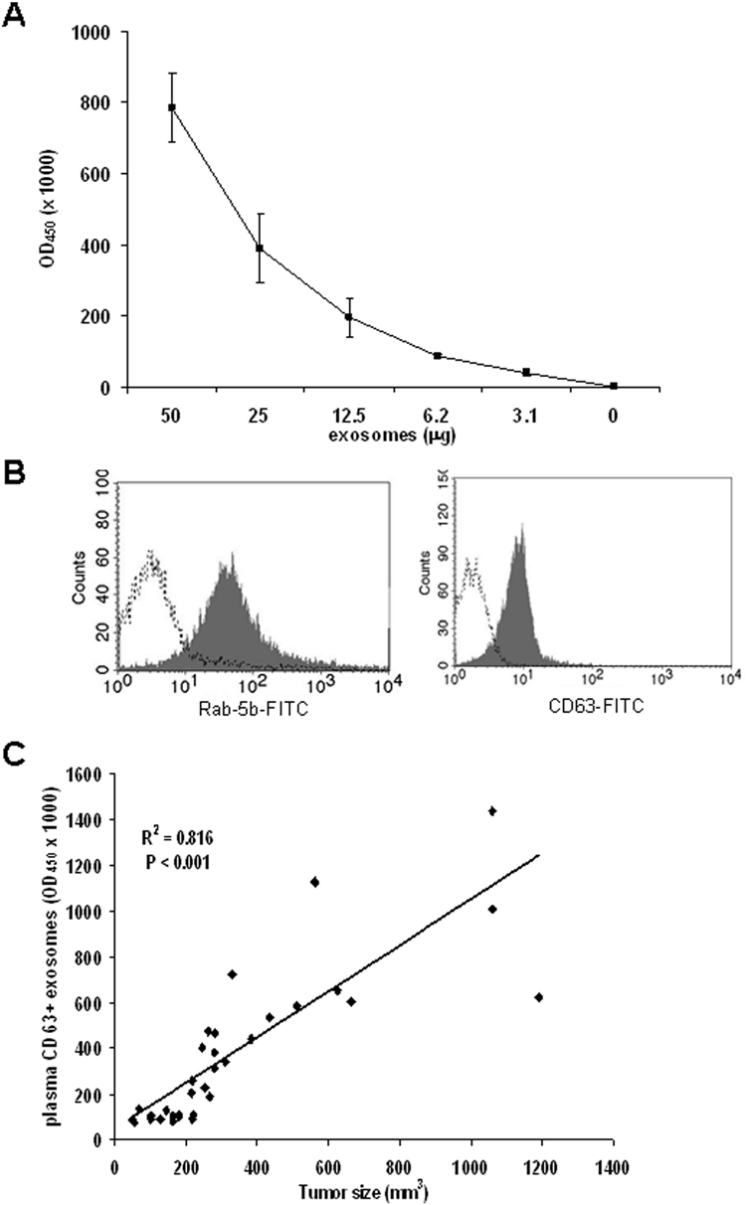
Detection of plasma exosomes of SCID mice engrafted with human melanoma. (A) Dose-escalation analysis of tumor exosomes purified from plasma of SCID mice engrafted with human melanoma cells by Exotest. (B) FACS analysis of Rab-5b and CD63 expression in exosomes purified from plasma of SCID mice engrafted with human melanoma cells (Me501). (C) Regression analysis between plasma levels of CD63+ exosomes and tumor size in 33 mice sacrificed 2–5 weeks after engraftment with Me501 cells.

In order to verify the possibility of designing a specific ELISA for the detection of human tumor-derived exosomes *ex vivo*, we purified plasma exosomes from SCID mice subcutaneously engrafted with human melanoma cells (Me501) 5 weeks after the engraftment. As for exosomes purified from cell culture supernatants, exosomes isolated from mice plasma were clearly and specifically detectable by ELISA (Figure 2A) and FACS (Figure 2B). Exosome preparations obtained from plasma of control SCID mice (not engrafted with human tumors) resulted in background optical densities comparable to blank samples (OD = 0.08±0.03), thus suggesting the absence of exosomes in the immunocompromised animals and that murine exosomes do not cross-react with human CD63 and Rab-5b.

The amount of plasma exosomes in mice engrafted with human melanoma was quantified in relation to tumor size. Melanoma-engrafted mice (n = 33) were sacrificed 2 to 5 weeks after the engraftment and plasma was obtained for exosomes isolation. Linear regression analysis showed a significant correlation between tumor size and levels of exosomes in plasma (Figure 2D). This results was confirmed also when Spearman correlation analysis was applied (Spearman coefficient 0.59, *P*<0.001). Interestingly, animals with very large tumors (>1000 mm^3^) showed amount of plasma exosomes not as high as expected. This might be due to the low vascularization and/or to the presence of necrotic area in the tumor that might not shed exosomes while increasing its size. Similar results were observed for the MeBS cell line (not shown). These data suggest that plasma exosomes quantification may represent a valuable biomarker to monitor tumor growth *in vivo*.

### Tumor exosomes express caveolin-1

Since exosomes are known to represent an important and specific route of intercellular communication [1], we reasoned that tumor-derived exosomes may differ from circulating exosomes in normal physiological conditions both in amount and proteins expression. Indeed, it has been recently reported that prostasomes (membrane vesicles secreted by prostate cancer cells) contain caveolin-1 (Cav1), a major component of caveolae [24] and that serum level of Cav1 is elevated in prostate cancer patients [25]. Moreover, we recently showed that Cav1 is highly expressed on vesicular structures of endolysosomal compartment in human melanoma cells [26].

**Figure 3:**
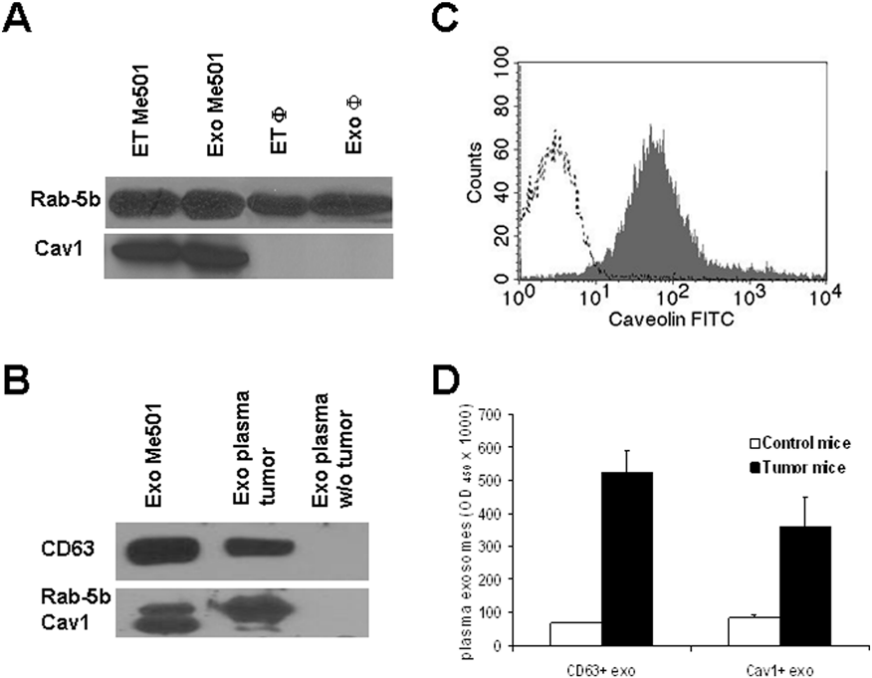
Characterization of caveolin-1 expression on exosomes. (A) Western blot analysis of Cav1 in cellular extracts and exosomes from human melanoma cells and macrophages (Φ). (B) Western blot analysis of CD63, Rab-5b and Cav1 in purified exosomes from Me501 cells, plasma of Me501-engrafted SCID mice and tumor-negative SCID mice. (C) FACS analysis of Cav1 expression on exosomes purified from plasma of Me501-engrafted SCID mice. (D) Plasma levels of CD63+ and Cav1+ exosomes from melanoma-bearing SCID mice sacrificed 5 weeks after engraftment.

First, we observed that Cav1 is strongly expressed on exosomes secreted by human melanoma cells *in vitro* while undetectable on both cellular extracts and exosomes from normal human cells such as for instance primary monocyte-derived macrophages (MDM) (Figure 3A), suggesting that Cav1 secreted in an exosome-embedded form may be a specific feature of melanoma cells, thus representing a potential marker for the ex-vivo analysis of tumor-derived exosomes. Therefore, we investigated the presence of Cav1 on exosomes obtained from plasma of SCID mice engrafted with melanoma tumors. Cav1 was detected in exosomes preparations derived from plasma of SCID mice engrafted with melanoma tumors by WB (Figure 3B), FACS (Figure 3C) and ELISA (Figure 3D) while Cav1 was undetectable in plasma-derived exosomes from control animals (Figure 3B, 3D). In agreement with previous results from melanoma and colo-rectal carcinoma (CRC) patients [10], [16], other tumor markers, such as MelanA/Mart-1 for melanoma and CEA for CRC, could be used for detecting the *in vivo* release of tumor exosomes in tumor-bearing SCID mice by Exotest, with results comparable with those obtained with Cav1. However, since melanoma may express heterogeneous or low amount of MelanA/MART-1, especially at metastatic levels [27], [28], and CEA is present mostly in soluble form in CRC patients serum, we considered Cav1 a more reliable and reproducible tumor marker, thus the Exotest was further developed with the inclusion of anti-Cav1-specific antibodies.

### Quantification and significance of exosomes in plasma of patients with melanoma

The data obtained in the human tumor-SCID mouse model prompted us to investigate whether the Exotest allowed the detection and characterization of exosomes purified from human plasma. Since human plasma may contain structures named microvesicles larger than exosomes, we first compared the Exotest reactivity of microvesicles and exosomes purified from the same plasma of 5 melanoma patients (n = 5). The Exotest revealed that while purified exosomes were captured and expressed both CD63 and Cav1, purified microparticles showed very low reactivity towards these two antigens (Figure S1).

**Figure 4:**
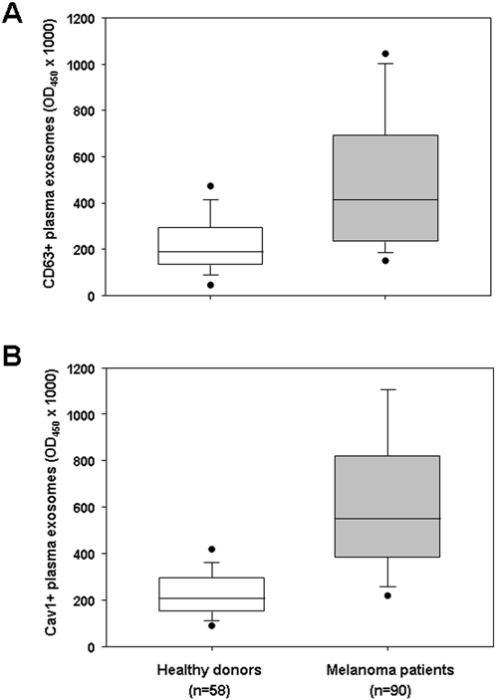
Quantification of exosomes in plasma from melanoma patients. Exosomes purified from plasma of healthy donors and melanoma patients were quantified by Exotest using as detection antigens CD63 (A) or caveolin-1 (B). Data are expressed as box plot representation: the horizontal and vertical lines in each box represent the median and the 25^th^–75^th^ percentiles, respectively; black dots represent outlier values. Differences between groups were evaluated by Mann-Whitney test and are reported in the text.

Exosomes were purified from plasma of tumor patients (n = 90) and healthy donors (n = 58) and quantified by Exotest based on the expression of CD63 and Cav1 (Table 1). As depicted in figure 4, the Exotest allowed the detection of exosome in plasma samples from both melanoma patients and healthy donors. However, plasma exosomes concentration was significantly higher in melanoma patients with respect to healthy individuals (*P*<0.001 for both CD63+ and Cav1+ exosomes). Interestingly, paired T-test showed that plasma levels of Cav1+ exosomes were significantly higher than levels of CD63+ exosomes in melanoma patients (*P* = 0.004).

**Table 1:** Plasma exosomes and serum LDH levels in the study population.

	CD63+ exo	Cav1+ exo	LDH
**Melanoma patients (n = 90)**	504±315	619±310	471±458
**Healthy donors (n = 58)**	223±125	228±102	360±345

CD63+ exo and Cav1+ exo are plasma exosomes (expressed as OD_450_×1000). Plasma LDH values are expressed as IU/L. Data are expressed as mean±SD.

In order to determine sensitivity and specificity of the Exotest based on the detection of the two exosomes protein markers, we calculated the cut-off both CD63 and Cav1-expressing plasma exosomes. The cut-off for CD63+ exosomes and Cav1+ exosomes was set at 2 times the standard deviations above the mean normal exosomes plasma level in the healthy controls, which was 473 and 432 (OD_450_×1000), respectively for CD63+ exosomes and Cav1+ exosomes. Accordingly, all samples with values above the cut-off were considered positive while samples with values below the cut-off were considered negative. The formulae for calculation of sensitivity and specificity are as follows. Specificity: (number of healthy controls with values below the cut-off / total number of healthy controls)×100. Sensitivity: (number of patients with values above the cut-off / total number of patients)×100. With these cut-off values, the specificity of the Exotest for detection of CD63+ plasma exosomes and Cav1+ plasma exosomes was 96.5% and 96.3%, respectively. However, while Exotest for CD63+ plasma exosomes showed a low sensitivity (43%) the Exotest for Cav1+ plasma exosomes had a higher sensitivity (69%).

These results suggest that i) circulating Cav1 may be associated to exosomes in melanoma patients and ii) quantification of plasma exosomes bearing Cav1 may be considered a useful tumor marker. In addition, we found that serum LDH did not correlate with either CD63+ or Cav1+ plasma exosomes while a significant correlation was observed between CD63+ and Cav1+ plasma exosomes (Spearman coefficient 0.32, P = 0.001). Moreover, the Exotest revealed the presence of tumor antigens, such as MART-1 or CEA in the plasma of melanoma patients (Figure S2), suggesting that plasma exosomes recovered from patients plasma are likely derived from tumor cells.

Most patients included in the analysis (82/90) were affected by advanced disease (stage III–IV). Besides six patients with very high serum LDH and poor prognosis, LDH levels at the time of plasma collection for exosome quantification were among the normal range (table 1). Nevertheless, a wide distribution of plasma levels of exosomes was detected by Exotest in all disease stages, suggesting that the variability in the amount of exosomes present in peripheral circulation of different patients may reflect diverse levels of tumor aggressiveness and may become a novel independent prognostic factor for melanoma. Other prognostic factors indicated by the American Joint Committee on Cancer (AJCC), including primary melanoma thickness and ulceration, number of metastatic lymph nodes, and site and number of distant metastases, might also correlate with exosomes serum content, as suggested by the data in mice engrafted with human melanoma, in which exosomes amount was directly associated with tumor burden. However, the relatively limited number of patients enrolled in this study does not allow presently to reach any statistical significance in the analysis.

### Use of whole plasma for exosomes quantification

**Figure 5:**
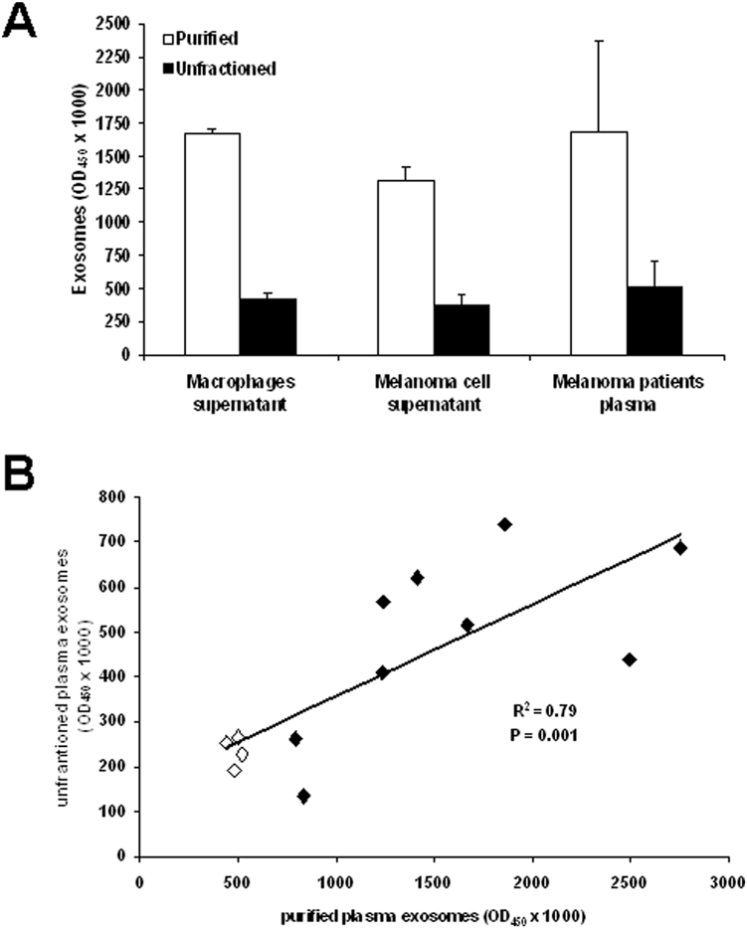
Exotest on unfractioned samples. (A) The amount of detectable exosomes was measured in purified exosomes (50 µg), unfractioned culture supernatants (50 µl) from human macrophages, melanoma cells and plasma from melanoma patients. Data are expressed as means±SD. (B) Regression analysis of plasma levels of CD63+ exosomes measured in purified or unfractioned plasma samples from both patients (n = 9, black diamonds) and healthy donors (n = 4, white diamonds). Exosomes levels are expressed as OD_450_×1000.

The potential applications of Exotest for clinical purposes prompted us to verify whether this assay could be utilized for exosomes detection in unfractioned biological fluids that would allow an easier and more reproducible analysis avoiding the steps of ultracentrifugation. Therefore, we compared the detection and quantification of CD63+ exosomes from unfractioned samples (cell culture supernatants from human macrophages and melanoma cells, and human plasma) and exosomes purified from the same samples. In order to increase the sensitivity of the test, for these specific experiments the HRP-conjugated Mab was incubated for 30 minutes instead of 15 minutes. As shown in figure 5A, the presence of exosomes from unfractioned macrophages and melanoma culture supernatants and plasma from 9 melanoma patients was detectable by Exotest. In addition, we performed the same analysis on plasma from 4 healthy donors and regression analysis on the total number of samples analysed (9 patients+4 healthy donors) showed a significant correlation between the two types of measures (Figure 5B). These results suggest the potential application of the Exotest in clinical settings using whole plasma and avoiding the complex and time consuming procedure of exosomes purification.

## Discussion

In this study we describe an in-house ELISA to detect and quantify exosomes from cell culture supernatants and human plasma, named Exotest. Exosomes are microvesicles produced by virtually all cells, but their secretion is known to be constitutively exacerbated in tumor cells, and several groups including ours, have reported the presence of exosomes of likely tumor origin in plasma and other biological fluids from cancer patients [10], [16]–[18], [29]. Although exosomes are implicated in a vast array of cellular functions, and have been considered a cell-free source of tumor antigens when secreted by cancer cells [11], [18], they have been lately hypothesized to foster immunosuppression in tumor-bearing hosts [3], [9], [12], [16], [30]. Recently, it was shown that the ability of serum exosomes from HNC patients to induce T-cell apoptosis correlated with disease activity and the presence of lymph node metastases [19]. Because of their potential involvement in promoting disease progression through a series of detrimental effects on tumor microenvironment, the possibility of quantifying exosomes in human plasma or serum is recently becoming an important issue. Such an assay would represent a fundamental tool for assessing the potential role of these microvesicles in cancer progression, and as a prognostic factor in the follow-up of cancer patients. Currently used methods for exosomes analysis and studies on their protein content include western blot, flow cytometry and mass spectrometry [1]. The quantitative method we describe here is instead based on ELISA-mediated detection of exosomes and represents to our knowledge the first report about an easy and reliable assay for exosome quantification. The proteins that are detected by our Exotest are not exosome-specific but are exclusively shared with cytoplasmic organelles, such as endosomes and lysosomes (Rab-5b and CD63), whose membranes are not recycled as for plasma membrane structures [1].

This excludes the possibility of detecting these proteins on plasma microparticles or debris derived from necrotic tumor cells, or in their soluble form. The assay we developed also included a tumor marker (caveolin-1) which allows the preferential detection of tumor-secreted exosomes. A series of comprehensive studies performed by different comparative methods (such as WB and FACS) and in different experimental conditions (supernatants from melanoma *vs* normal cells, and plasma from normal *vs* melanoma-engrafted SCID mice) proved the reliability of the Exotest. Moreover, we found that cell lines of other tumor histotypes (osteosarcoma and CRC) secreted exosomes expressing variable levels of CD63 and Cav1 (Figure S2), in line with the reported cellular expression of Cav1 in these cancers [31], [32]. Using this assay, we observed that significantly increased amounts of exosomes expressing tumor markers such as caveolin-1 are present in plasma from melanoma patients with respect to healthy individuals. Interestingly, the wide distribution of Cav1+ plasma exosomes within the patients' population suggests the potential use of exosomes in plasma as prognostic marker. In fact, plasma levels of Cav1-expressing exosomes were significantly decreased in patients undergoing chemotherapy with respect to patients untreated at time of sampling (data not shown). Since this was a cross-sectional analysis of patients undergoing diverse chemotherapy regimens, longitudinal studies on patients undergoing strictly controlled chemotherapy protocols will better define the importance of this observation. It should be underlined that exosomes are also detected in normal subjects, being secreted also by normal cells of different organs, including blood cells. However, the levels of exosomes quantified in plasma of melanoma patients are significantly above the cut-off level calculated from healthy donors, suggesting a good level of sensitivity and a high specificity of detection. Recently, it has been reported that plasma exosomes of melanoma patients promote the generation of suppressive myeloid cells [10] and the induction of a series of different functional defects in activated T cells [9], [16], [33], suggesting that tumor cells may use exosomes to damage the immune system without a direct interaction with immune cells. Moreover, it is progressively emerging that these vesicles can be used by tumor cells as a non-cellular tool for microenvironment remodelling, promotion of neo-angiogenesis, and sustainment of their own growth through autocrine loop [3], [12]. Interestingly, human prostate cancer cells secreting caveolin-1 induced tumor growth of caveolin-1 negative tumor cells *in vivo* through the release of caveolin-1 associated to lipoprotein particles [34]. The selective and/or preferential expression of caveolin-1 on exosomes from tumor patients may thus represent an important marker of malignant progression and deserves further investigation about its possible application as a screening method in tumor patients. Notably, this is the first evidence that caveolin-1 is expressed on exosomes released by human tumors.

An alternative and more intriguing hypothesis is that exosomes could be a hallmark of more aggressive tumors, and thus high exosomes plasma levels could identify patients with unfavorable prognosis despite early disease stage. Indeed, the unique biochemical properties of these organelles and the peculiar lipid composition of their membranes may determine their long-term persistence in plasma also in patients whose tumor has been surgically removed [30]. Because of the lack of a reliable quantitative assay, no study has so far addressed whether the amount of exosomes in plasma may associate with a different disease course in cancer patients. This is an even more important issue in melanoma, which is a rather heterogeneous disease, with subsets of patients undergoing unexpectedly poor prognosis despite the presence of good prognostic factors and vice versa.

Melanoma is still one of the cancers in which a soluble tumor marker in support of prognosis and treatment evaluation has not yet been identified [35]. Our results suggest that an exosome-specific ELISA may be used for detection and quantification of circulating exosomes in melanoma patients. Moreover, the test offers the possibility of detecting different proteins in plasma exosomes preparations, with the potential application to specific type of tumor patients. We reckon that longitudinal clinical studies on larger cohorts and standardization of the method described are to be performed in order to evaluate whether plasma exosomes quantification and characterization may represent an independent prognostic factor for melanoma patients and possibly for patients carrying other types of cancers. Nevertheless, this assay may help exploring a rather new field in cancer research for the identification of novel prognostic tools for cancer.

## Methods

### Cell culture

We used two human metastatic melanoma cell lines (Me501 and MeBS) obtained from metastatic melanoma lesions of patients (Istituto Nazionale Tumori, Milan, Italy). Tumor cells were negative for Mycoplasma contamination as routinely tested by PCR (Venor®GeM, Minerva Biolabs, Germany). Cell lines were cultured in RPMI 1640 medium supplemented with antibiotics and 10% fetal calf serum (FCS) (Invitrogen, Milan, Italy) previously depleted from bovine microvesicles by ultracentrifugation (100,000×g for 90 minutes). Human monocytes-derived macrophages (MDM) were obtained from buffy coats of healthy blood donors by using CD14 magnetic beads ((Miltenyi Biotec, Germany) and GM-CSF (500 U/ml) for 5 days in culture.

The osteosarcoma (SaOS-2) and colon carcinoma (Colo 1869) cell lines were a kind gift of Dr. Maccalli (San Raffaele Scientific Institute, Milano) and Dr. Serra (Istituti Ortopedici Rizzoli, Bologna).

### Exosomes purification from cell culture supernatants and plasma

Supernatants from human melanoma cell lines were harvested from 72 hs 70–75% confluent cell cultures in 175 cm^2^ flasks and were isolated as previously described [36], [37]. Briefly, after centrifugation of cells at 300 *g* for 10 minutes, supernatants were centrifuged at 1,200 *g* for 20 minutes followed by 10,000 *g* for 30 minutes. Supernatants were filtered using a 0.22 µm filter (Millipore Corp., Bedford, MA) and centrifuged at 100,000 *g* for 1 h in a Beckman ultracentrifuge (Beckman Coulter) in order to pellet exosomes. After 1 wash in a large volume of phosphate-buffered saline (PBS), exosomes were resuspended in PBS (50–100 µl) or in lysis buffer, and stored at −80°C for experimental analysis.

In order to obtain exosomes from plasma samples, EDTA-treated blood from SCID mice engrafted with human melanoma or plasma from tumor patients and healthy donors were centrifuged at 400 *g* for 20 minutes. Plasma was then collected and stored at −70°C until analysis. Plasma samples were subjected to the same centrifugal procedure described above to isolate exosomes. In addition, for some samples the pellet recovered after the centrifugation at 10,000 *g* for 30 minutes representing microparticles was collected and analysed.

### ELISA for exosomes detection

Ninety-six well-plates (Nunc, Milan, Italy) were coated with polyclonal 4 µg/ml anti-Rab-5b antibody (clone A-20, Santa Cruz) in a volume of 100 µl/well of carbonate buffer (pH 9.6) and incubated overnight at 4°C. After 3 washes with PBS, 100 µl/well of blocking solution (PBS containing 0.5% BSA) were added at room temperature for 1 hour. Following 3 washes in PBS, exosomes purified from cell culture supernatants or from plasma were added in a final volume of 50 µl and incubated overnight at 37°C. After 3 washes with PBS, anti-CD63 Mab (clone H5C6, Pharmingen) or anti-caveolin-1 Mab (clone 2297, Pharmingen) diluted 4 µg/ml were added and incubated for 1 hour at 37°C. After 3 washes with PBS, the plate was incubated with 100 µl of HRP-conjugated anti-mouse antibody (Pierce, Milan, Italy) diluted 1∶50,000 in blocking solution for 1 hour at room temperature. After the final 3 washes with PBS, the reaction was developed with POD for 15 min (Roche Applied Science, Milan), blocked with H_2_SO_4_ and optical densities were recorded at 450 nm.

### Flow cytometry analysis of exosomes

Exosome preparations (5–10 µg) were incubated with 5 µl of 4-µm-diameter aldehyde/sulfate latex beads (Interfacial Dynamics, Portland, OR) and resuspended into 400 µl PBS containing 2% FCS. Exosomes-coated beads (20 µl) were incubated with the following antibodies: anti-CD63-FITC (Pharmingen), anti-CD81-PE (Pharmingen), anti-Rab5b (Santa Cruz), anti-caveolin-1 (clone N-20, Santa Cruz) for 30 minutes at 4°C, followed, when needed, by incubation with PE- or FITC-conjugated secondary antibody and analyzed on a FACSCalibur flow cytometer (BD Biosciences).

### Western blot analysis of exosomes

Purified exosomes or cells were treated with lysis buffer (1% Triton X-100, 0,1% SDS, 0.1 M Tris HCl, pH 7) and protease inhibitors (Sigma) and protein concentration was determined by Bradford microassay (Bio-Rad Laboratories, Hercules, CA). Proteins were separated on 10% SDS-PAGE gel and transferred to nitrocellulose membranes. Membranes were blotted with antibodies to CD63 and Lamp-1 (Mabs, Pharmingen), Rab-5b and caveolin-1 (polyclonal antibodies, Santa Cruz), incubated with appropriate HRP-conjugated secondary antibodies (Amersham Pharmacia) and visualised by enhanced chemiluminescence (Pierce).

### Human tumor SCID-mouse model

CB.17 SCID/SCID female mice (Harlan, Milano, Italy) were used at 4–5 weeks of age and kept under specific pathogen-free conditions. Animal care was conformed to the European Council Directive 86/609/EEC and the study was approved by the institutional review board. Mice were injected subcutaneously into the right flank with 2.5×10^6^ human melanoma cells and tumour weight was measured by a caliper with the formula: Tumor size (mm^3^) = length×width^2^ /2 [38]. Five-hundred µl of blood from tumor-engrafted mice were collected from different animals sacrificed at different time-points during tumor growth.

### Melanoma patients

Human plasma samples were collected from EDTA-treated whole blood from patients with melanoma (n = 90) attending the Istituto Nazionale dei Tumori (Milan) and the Ospedale Le Scotte (Siena) and age and sex-matched healthy donors (n = 58) and stored at −70°C until analysis. The study was approved by the local ethical committees and patients gave written informed consent to participate.

### Statistical analysis

Statistical analyses were performed by using the software SigmaStat (SPSS Inc.). Differences between melanoma patients and healthy donors were analysed by Mann-Whitney test, Wilcoxon signed rank paired test or student T test as appropriate. Correlation between variables was assessed by Spearman rank test or regression analysis. Data in the text are expressed as mean±SD. All P values reported are two-sided.

## Supporting Information

Figure S1 (0.16 MB TIF)

Figure S2 (0.11 MB TIF)
