# Supplementary figures and images for: High Levels of Exosomes Expressing CD63 and Caveolin-1 in Plasma of Melanoma Patients
Source: PLoS One. 2009 Apr 17;4(4):e5219. doi: 10.1371/journal.pone.0005219 (PMC2667632; doi:10.1371/journal.pone.0005219)

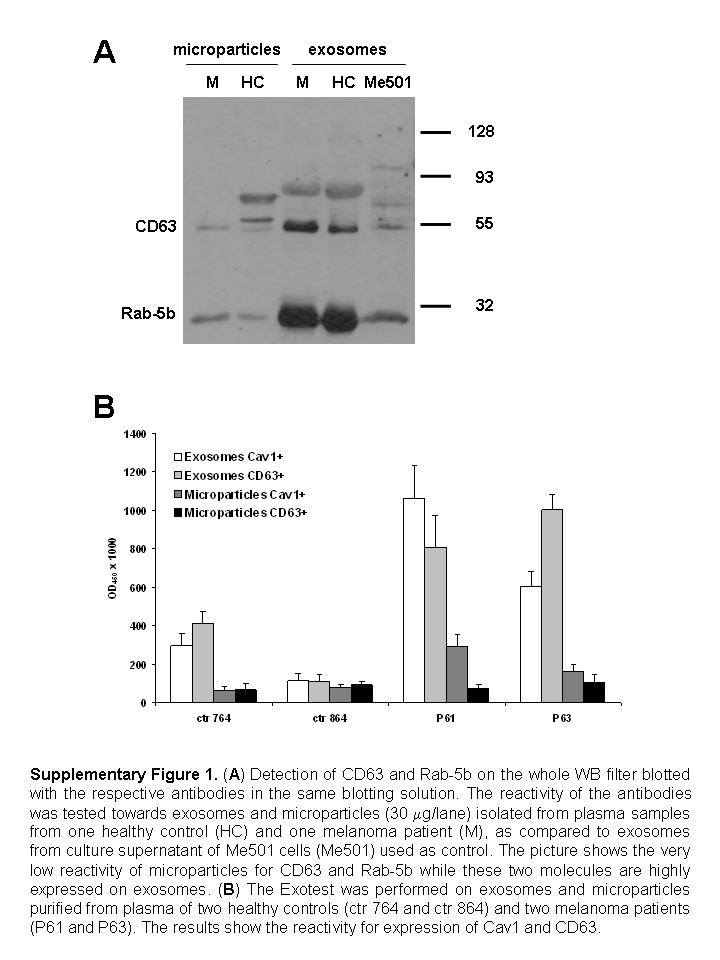

Supplement: Figure S1 — (0.16 MB TIF) [file pone.0005219.s001.tif]

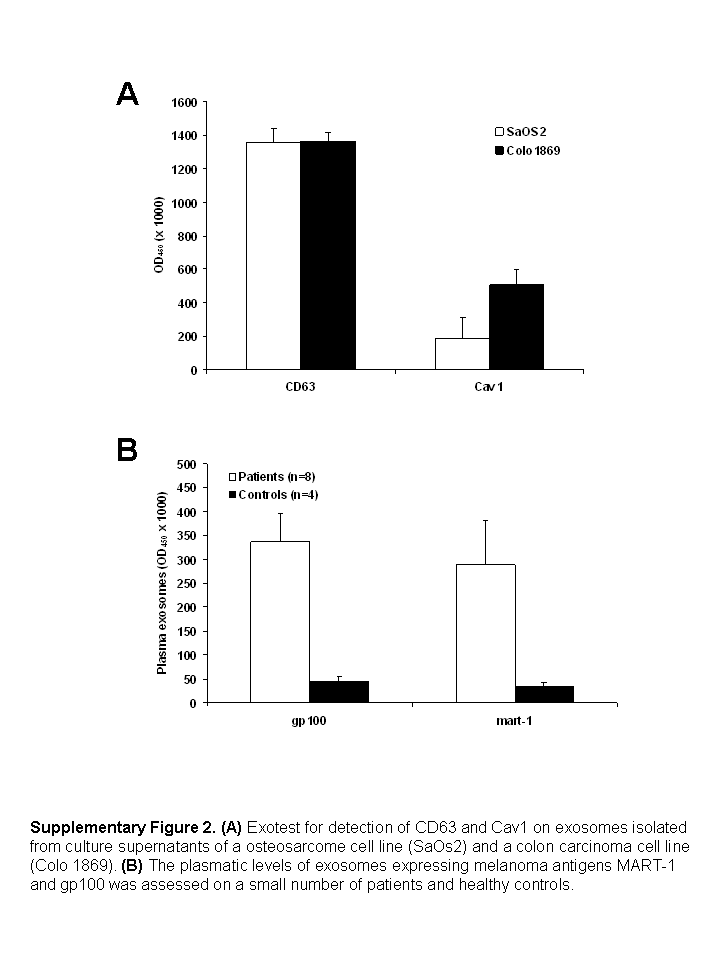

Supplement: Figure S2 — (0.11 MB TIF) [file pone.0005219.s002.tif]
